# Gene Regulatory Networks Elucidating Huanglongbing Disease Mechanisms

**DOI:** 10.1371/journal.pone.0074256

**Published:** 2013-09-25

**Authors:** Federico Martinelli, Russell L. Reagan, Sandra L. Uratsu, My L. Phu, Ute Albrecht, Weixiang Zhao, Cristina E. Davis, Kim D. Bowman, Abhaya M. Dandekar

**Affiliations:** 1 Department of Plant Sciences, University of California Davis, Davis, California, United States of America; 2 Dipartimento di Sistemi Agro-ambientali, Università degli Studi di Palermo, Palermo, Italy; 3 U.S. Horticultural Research Laboratory, U.S. Department of Agriculture, Agricultural Research Service, Fort Pierce, Florida, United States of America; 4 Department of Mechanical and Aerospace Engineering, University of California Davis, Davis, California, United States of America; The Ohio State University/OARDC, United States of America

## Abstract

Next-generation sequencing was exploited to gain deeper insight into the response to infection by *Candidatus liberibacter asiaticus* (CaLas), especially the immune disregulation and metabolic dysfunction caused by source-sink disruption. Previous fruit transcriptome data were compared with additional RNA-Seq data in three tissues: immature fruit, and young and mature leaves. Four categories of orchard trees were studied: symptomatic, asymptomatic, apparently healthy, and healthy. Principal component analysis found distinct expression patterns between immature and mature fruits and leaf samples for all four categories of trees. A predicted protein – protein interaction network identified HLB-regulated genes for sugar transporters playing key roles in the overall plant responses. Gene set and pathway enrichment analyses highlight the role of sucrose and starch metabolism in disease symptom development in all tissues. HLB-regulated genes (glucose-phosphate-transporter, invertase, starch-related genes) would likely determine the source-sink relationship disruption. In infected leaves, transcriptomic changes were observed for light reactions genes (downregulation), sucrose metabolism (upregulation), and starch biosynthesis (upregulation). In parallel, symptomatic fruits over-expressed genes involved in photosynthesis, sucrose and raffinose metabolism, and downregulated starch biosynthesis. We visualized gene networks between tissues inducing a source-sink shift. CaLas alters the hormone crosstalk, resulting in weak and ineffective tissue-specific plant immune responses necessary for bacterial clearance. Accordingly, expression of WRKYs (including WRKY70) was higher in fruits than in leaves. Systemic acquired responses were inadequately activated in young leaves, generally considered the sites where most new infections occur.

## Introduction

Huanglongbing (HLB) or “citrus greening” is the most destructive citrus disease worldwide [1] and no cure is currently available. It is caused by three species of Gram-negative, phloem-inhabiting α-proteobacteria, *Candidatus Liberibacter* spp. “*Ca. L. asiaticus*” (CaLas), “*Ca. L. africanus*”, and “*Ca. L. americanus*”. The pathogen is transmitted by two species of phloem-feeding citrus psyllids, *Diaphorina citri* and *Trioza erytreae*. The disease affects most citrus species although different responses have been observed for different genotypes and species in the *Citrus* genus [2]–[4]. HLB symptoms include yellow shoots, blotchy mottled leaves, and lopsided fruits with poor and inverted coloration and aborted seeds. Moreover, swelling of middle lamella between cell walls surrounding sieve elements, starch accumulation in leaves, and phloem damage are observed [5], [6]. Disease incubation times are long and quantitative PCR detection is unreliable before symptoms appear [7]. Secondary infection spreads quickly because the insect vector shows high incidence of CaLas before symptomatic plants can be discerned.

Although substantial research efforts have been made to detect the pathogen with quantitative RT-PCR [7] or microarrays [8], little is known about the physiological mechanisms of this disease. Efforts have been undertaken to culture the bacteria *in vitro* [9]. The genome sequence of CaLas was obtained using a metagenomic approach from plant vascular tissues [10] and infected psyllids [11]. Since no toxins, cell wall degrading enzymes, or specialized secretion systems were found in the genome, it is believed that the disease results from host metabolic imbalances due to nutrient depletion or interference with nutrient transport [12]. Microarrays have been used to characterize some host responses to HLB infection in mature leaves [6], [12] and to discover key genes in tolerant and susceptible citrus genotypes [3], [13], [14]. Recently, the isobaric tags for relative and absolute quantitation (iTRAQ) technique was used to characterize proteome changes in CaLas-inoculated citrus, identifying potential targets of early infections [15]. Next-generation sequencing technologies can enable a deeper analysis of the RNA population than microarrays, including rare and unknown transcripts, offering a more precise and accurate picture of the transcriptome [15], [16]. Metabolomics has proven effective for studying metabolic changes [17] in response to agronomic treatments and environmental stresses including CaLas infection [18]–[21].

Previously we presented an RNA-Seq transcriptome analysis of mature fruit of infected field trees at different disease stages [22]. Here, we expand this analysis to source and sink tissues (fruits and leaves) of naturally infected trees in orchards, seeking to determine the gene regulatory networks underlying the metabolic disorder of the disease and to dissect pathogen-induced dysfunctions in source-sink relationships and hormone crosstalk. We have dissected the citrus host responses, integrating different functional methods including principal component analysis (PCA), gene set and pathway enrichment, and protein-protein network analyses to identify key genes that may potentially serve as targets for short-term therapeutic treatments.

## Results

### Transcriptome and functional analysis

In addition to the previous transcriptome data from the mature fruit peel [21], RNA-Seq was performed on other three tissues from each of four HLB phenotypes (Table 1), RNA-Seq data from all four tissues were analyzed together using the new reference dataset [23]. For the 16 cDNA libraries, a total of 889 million 85 bp paired-end raw reads were obtained with the Illumina Genome Analyzer II. These reads were trimmed and aligned to the *Citrus sinensis* genome produced by the US Department of Energy Joint Genome Institute (http://www.jgi.doe.gov) in collaboration with the user community (http://www.phytozome.net/citrus). Expressed genes and transcript isoforms were identified and annotated on the *C. sinensis* genome v.1 assembly consisting of 12,574 scaffolds [23]. A list of the differentially regulated transcripts with corresponding *Arabidopsis* orthologs was obtained for three pairwise comparisons for each tissue (Dataset S1). In separate principal component analysis and differential expression analysis of count data, three clusters of overall expression profiles were found among the 16 sample types, where mature and immature fruits comprise two clusters, and all leaf samples comprise the third cluster (Figure S1). Due to potential confounding environmental and agronomic disparities between orchards, our analysis primarily focuses on trees from the same location, comparing apparently healthy (AH) and symptomatic (SY) samples in each of the four tissues. AH trees were growing at the same location as the SY trees, showed no symptoms, but were PCR-positive for the pathogen at the time of sampling.

**Table 1:** Abbreviations used for 16 sample types.

disease status	AH	apparently healthy
	AS	asymptomatic
	SY	symptomatic
	CO	HLB-free orchard (control)
tissue type	IF	immature fruits
	MF	mature fruits
	YL	young leaves
	ML	mature leaves

**Figure 1:**
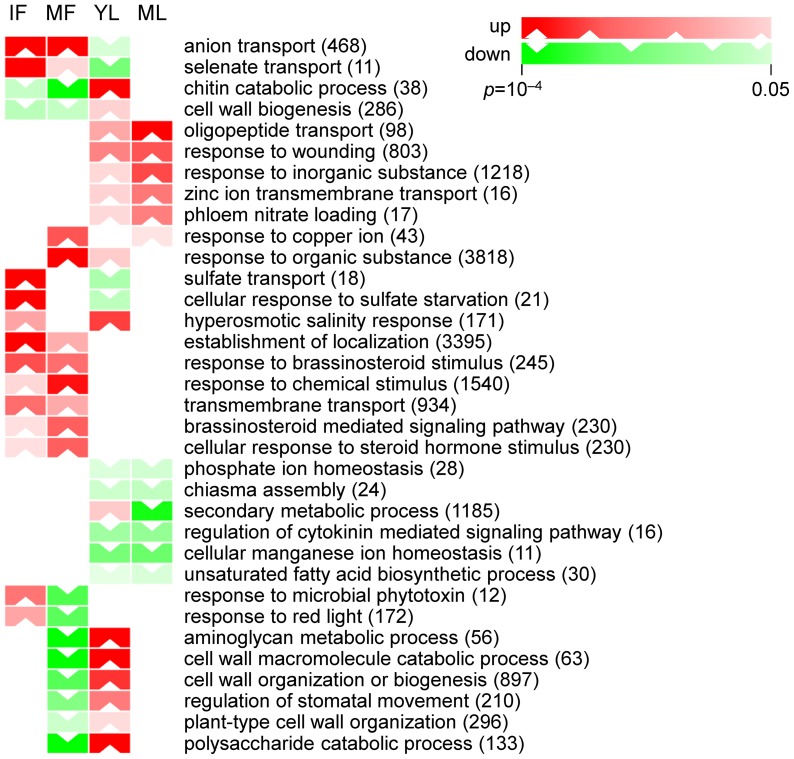
Gene Set Enrichment Analysis based on sparse principal component analysis (sPCA). GO terms representing differentially regulated functional classes of genes in at least two of the four tissues (*p*<0.05). Color values correspond to *p*-values, as indicated by color bars. Total genes in each gene set (more than ten genes) are given in parentheses. Each column represents one of four tissues: IF, immature fruits; MF, mature fruits; YL, young leaves; ML, mature leaves. Complete GSEA results, including sets with <10 genes, and enriched in one tissue only, are given in Dataset S2.

Gene set enrichment analysis (GSEA), based on a sparse principal component analysis (sPCA) technique, clearly showed that transport-related pathways in leaves and fruits were affected differently by HLB (Figure 1). In fruits, Gene Ontology (GO) terms related to ion transport (particularly sulfate, selenate, and copper) were strongly upregulated. In mature infected leaves, more transcripts related to oligopeptide, zinc, and nitrate transport were upregulated. Genes related to cell wall organization, biogenesis, and catabolism were upregulated in young leaves and downregulated in mature fruits. In young leaves, aminoglycan, polysaccharide, and chitin catabolism were induced by the disease. Cell wall modification and restructuring are key processes in signal transduction of biotic responses. They would logically be more affected in newly infected tissues where pathogen load could be higher.

Brassinosteroid signaling components were upregulated in fruits. A complete list of significantly enriched GO terms in the AH vs SY comparison is presented in Dataset S2. In pathway enrichment analysis, sucrose and starch metabolism were the most significantly differentially regulated pathways in all four tissues (Table 2). Secondary metabolism was more affected by the disease in immature fruits while primary metabolism was significantly regulated in ripe ones.

**Table 2:** Differentially regulated* pathways in response to HLB disease.

	Fruits		Leaves	
Pathway	Imm.	Mature	Young	Mature
Starch and sucrose metabolism	9.5*10^–7^	0.01	0.04	2*10^–4^
Phenylpropanoid biosynthesis	0.003	n.s.	0.03	0.01
Indole, ipecac alkaloid biosynth.	0.03	n.s.	n.s.	n.s.
α-Linolenic acid metabolism	0.03	0.02	n.s.	n.s.
Anthocyanin biosynthesis	0.04	n.s.	n.s.	n.s.
Metabolism of xenobiotics	0.04	n.s.	n.s.	n.s.
Carotenoid biosynthesis	0.05	n.s.	0.06	n.s.
Phenylalanine metabolism	0.05	n.s.	n.s.	n.s.
Carbon fixation	n.s.	0.004	0.004	0.09
Glycerolipid metabolism	n.s.	0.01	0.01	0.04
Glycolysis/Gluconeogenesis	n.s.	0.03	n.s.	0.05
Galactose metabolism	n.s.	0.04	n.s.	n.s.
Pentose, glucuronate interconv.	n.s.	0.07	n.s.	n.s.
Riboflavin metabolism	n.s.	0.1	n.s.	n.s.
Indole and ipecacalkaloid biosynthesis	n.s.	n.s.	n.s.	0.02

* Based on genes with log fold ratio <−1.5 and >1.5 between AH and SY samples; *p*-values are shown for GSEA using Pathexpress web tool, with *p*<0.1 considered to be significantly HLB-regulated; n.s.  =  not significant.

The pattern of gene expression in mature leaves was compared with previous studies on responses to biotic stresses such as citrus bacterial canker disease (CBCD) [24] and citrus tristeza virus (CTV) [25]. Interestingly, host sucrose and starch metabolism were altered in both HLB and CBCD while CTV affected other pathways such as pentose phosphate, glutathione, ascorbate, and aldarate metabolism (Table S1). Comparing GSEA results between AH or AS and SY categories reveals citrus responses as symptoms appear. Interestingly in leaves, genes related to starch were mainly upregulated while minor carbohydrate genes were downregulated (Figure S2). MapMan metabolic overviews show expression changes in specific genes (Figs. S3, S4, S5).

### HLB alteration of fruit transcriptome

**Figure 2:**
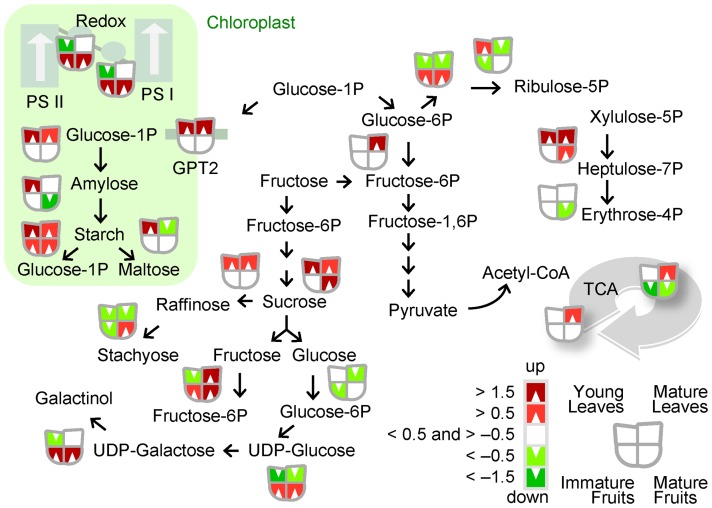
HLB-regulation of photosynthesis and carbohydrate metabolism. Overview of changes induced by HLB in the expression of genes affecting photosynthesis and small carbohydrate metabolism (AH vs. SY samples; see Table 1 for a key to abbreviations for sample types).

Several HLB-regulated genes were involved in primary metabolism: invertase (sucrose degradation), ADP glucose pyrophosphorylase large subunit (starch biosynthesis), α- and β-amylase (starch metabolism), glucose-6-phosphate dehydrogenase 1 (oxidative pentose phosphate), galactinol synthase, and stachyose synthase (raffinose metabolism; Figure 2). Interestingly, several genes involved in lipid metabolism, ammonia metabolism, sulfate assimilation, C1-metabolism, and nucleotide metabolism were overexpressed in response to HLB. Secondary metabolism was highly affected by the disease, with HLB-regulated genes involved in terpene metabolism, flavonoids, and phenylpropanoids (Figure S3).

### HLB alteration of leaf trancriptome

In young leaves, the site of most primary infections, we observed a general downregulation of genes involved in photosynthesis, the Calvin cycle (fructose bisphosphate aldolase, glyceraldehyde-3-phosphate dehydrogenase, and rubisco activase), and photorespiration (glycine cleavage H system, glycerate dehydrogenase). Conversely, genes involved in sucrose and starch metabolism were upregulated: α-glucan water dikinase, α-glucan phosphorylase 2, invertases, glucose-1-phosphate adenylytransferase 3 (APL3), and starch branching enzyme. Several genes involved in cell wall modification and degradation were induced by HLB, as were others involved in flavonoid, terpenoid, phenylpropanoid, and amino acid metabolism (aspartic acid, glutamate, tryptophan, and tyrosine metabolism; Figure S3).

In mature leaves, HLB disease severely affects sucrose and starch metabolism, the Calvin cycle, glycolysis, pentose phosphate, and mitochondrial electron transport (Figs. S3, S4). Secondary metabolism was also affected, especially upregulation of anthocyanin-5-aromatic-acyltransferases, naringenin chalcone synthase, UDP-glucosyl transferases, flavonoid-3-monooxygenase, myrcene synthase, laccase 7, and O-methyltransferase genes.

### Light reaction genes

Most photosynthesis-related genes were downregulated in young infected leaves, including those of light harvesting complex B5 (LHCB5) and B2.1 (LHCB2.1) (Figure S3). Conversely, in fruit, several genes were upregulated: photosystem I light harvesting complexes 1 and 2, photosystem I PSI-N, chloroplast, PSI-N, (PSAN), photosystem I P, photosystem I subunit O, and cytochrome b6f complex subunit (petM).

### Transcription factors

HLB disease drastically affects expression of important classes of transcription factors (TFs) in both leaf and fruit tissues. In young leaves, several AP2-EREBP, bHlH proteins, MYB domain factors, zinc finger C2H2-type factors, and WRKY members were upregulated. Conversely, CAL1, AGL14, LBD37, ERF23, SHN2, and ERF26 TFs were less abundant when symptoms appeared. In mature leaves, HLB affected transcription of genes belonging to several families: AP2-EREBP (Rap 2.6L, CRF4), MYB (MYB62), bZIP (ZIP5, ABI5), AS2 (LBD11 and LBD25), bZIP (NTT, C2H2-type), ABI3/VP1, CCAAT-HAP2, and WRKY (WRKY23, WRKY31, WRKY42, and WRKY47).

In immature fruit, changes in genes encoding CCAAT box binding factor, trihelix, nucleosome assembly, and G2-like proteins were observed. Several WRKY transcripts were more abundant at the symptomatic stage: WRKY6, WRKY18, WRKY50, WRKY53, WRKY54, and WRKY70.

### HLB regulation of hormone crosstalk

**Figure 3:**
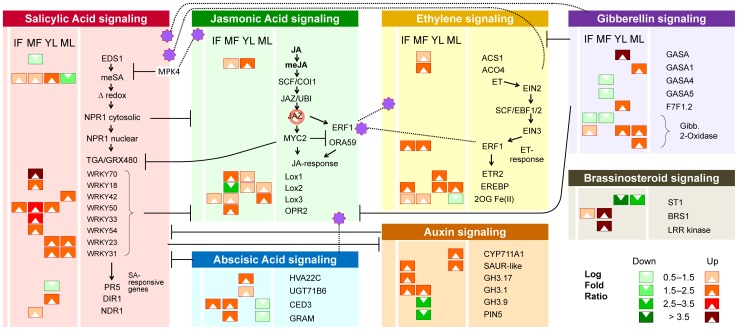
HLB-induced modulation of hormone-mediated immune responses. Four AH vs. SY tissues were analyzed. Regulatory interactions between pathways [30] are shown. See Table 1 for a key to abbreviations for sample types.

Hormone signaling pathways strongly affect the timing and intensity of disease responses in plants. The overall hormone crosstalk network was strongly affected by HLB disease (Figure 3). The methylsalicylic acid transferase gene, coding for the long distance signal for the salicylic acid (SA) mediated defense response, was upregulated in young, HLB-infected leaves and downregulated in mature ones. Unchanged expression of NPR1 (nonexpressor of PR genes1) suggested that SA signaling was insufficiently activated in response to HLB. SA-mediated defense response-related genes were not activated, except for PR5 and DIR1. More WRKY TFs were induced in fully ripe fruits than in young leaves. The jasmonic acid carboxyl methyltransferase gene, responsible for long distance signaling in the ISR (induced-systemic response) was induced in young leaves and to a lesser degree in mature fruits. Several jasmonic acid metabolism genes were upregulated in fruits: lox1, opr2, jaz10, and jar1. In ripe infected fruits, several genes involved in ethylene biosynthesis and response were affected: ACO4, ACS1, ERF2, and ERF6. ABA, auxin, gibberellin, and brassinosteroid pathways were affected by HLB in leaf and fruit tissues (Figure 3). Interestingly, auxin-related genes were highly induced in immature fruit. Gibberellin-related genes showed an opposite pattern of expression: mainly downregulated in fruits, but upregulated in leaves. The expression of key brassinosteroid genes was affected in both leaf and fruit tissues: BRS1 was upregulated in mature fruits while ST1 was downregulated in young and mature leaves.

### Amino acid metabolism

Amino acids play important roles in plant responses to stresses. Of the ten gene families of interest, six were not regulated by HLB disease in any of the four tissues (Figure S6). In addition, overall downregulation of arginine transport genes was seen in young leaves (Dataset S2).

### qRT-PCR validation

Gene expression analyses using qRT-PCR were performed to corroborate RNA-Seq data (Dataset S3). In young leaves, acidic cellulose and terpene synthase cyclase genes were significantly induced in SY tissues. Interestingly, several ethylene-responsive transcription factors and auxin-related genes (GH3.1 and GH3.4) were induced in fruits. Starch-related genes were induced in leaves while invertase was upregulated in infected fruits. WRKY70 transcription factor was highly induced in mature fruit peel. The glucose-phosphate transporter (GPT) was strongly upregulated in SY leaves. In mature fruits, several genes were induced at the AS stage: MYB factors, PDR11 transporter, and ring family protein.

### Protein-protein network analysis

**Figure 4:**
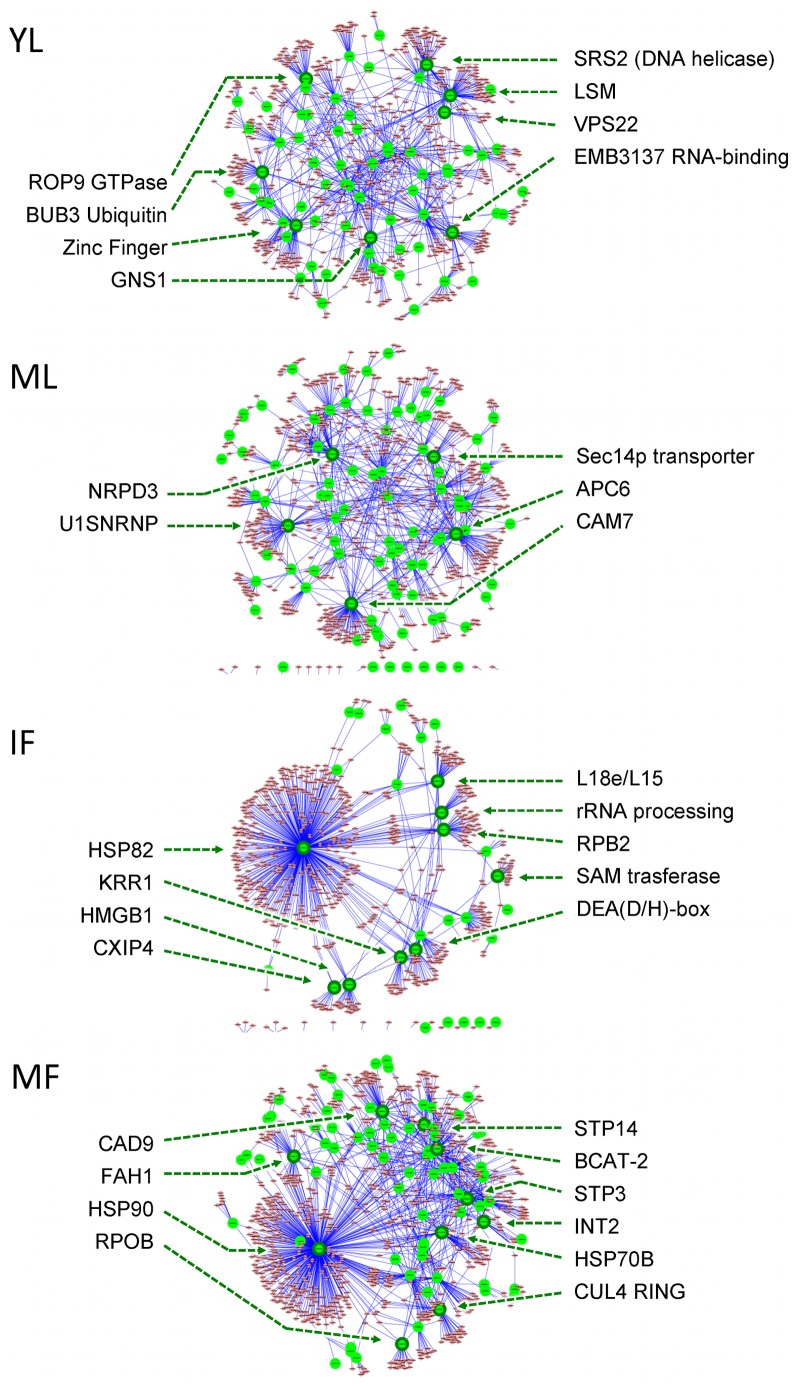
Predicted protein-protein interaction networks of Citrus responses to HLB disease. Four citrus tissues were analyzed using the dataset of HLB-regulated genes in SY versus AH samples based on an *Arabidopsis* knowledgebase. HLB-regulated proteins are represented by the larger nodes. (Fold ratio >1 or <−1). See Table 1 for a key to abbreviations for sample types.

A protein-protein interaction network (PPI) was deduced between proteins encoded by HLB-regulated genes and their predicted interactions (Figure 4; Figure S7). The overall PPI networks differed markedly between the four tissues. Different developmental stages within same tissue were more similar (Dataset S4). In young leaves, several small hub proteins involved in transcription and DNA replication were observed. Several proteins involved in signaling (i.e. CAM7) and sugar transport were observed only in mature leaves. HLB regulation of HSP82 drastically affected the fruit PPI network at both developmental stages. At the immature stage, several HLB-regulated proteins were involved in transcription: L18e/L15, RPB2, DEA (D/H)-box, KRR1, and SAM transferase. When fruit ripened, several interactive proteins were involved in sugar transport (STP3, STP14, and INT2).

## Discussion

Previous studies have focused on mature leaf tissues, using plants artificially infected with CaLas, or on mature fruit in orchard conditions [21]. These did not examine the link between host responses in source and sink tissues, where mature leaves comprise the source, and young leaves along with immature fruits comprise the sink. Although these studies identified HLB-regulated genes involved in key host pathways, the experiments were conducted under a variety of conditions: graft or natural infections, greenhouse or orchard conditions, which may undermine the validity of conclusions regarding mechanisms of symptom appearance at whole plant level. The results in the present study were derived from a unified transcriptome analysis in different plant organs (leaves and fruits) at different developmental stages (immature and mature) in naturally infected trees.

Physiological mechanisms of HLB disease are poorly understood. Since no toxins, extracellular degrading enzymes, or specialized secretion systems were found in the CaLas genome [11], a pathogen-induced host source-sink metabolic imbalance is likely to be the main cause of disease symptoms.

Transcriptomic analysis of infected plants in orchards allows observation of host responses to psyllid-transmitted infections under natural conditions to augment controlled environment experiments. To gain insight into the disease mechanisms at a deeper level than previous studies, this study functionally analyzed the transcriptome of four tissue types representing the main metabolic changes (Figure 3, Figure S8). Using integrated methods of analysis (PCA, GSEA, pathway enrichment, MapMan functional categorization, and PPI network analysis), we dissected disease mechanisms, focusing on key HLB-regulated pathways such as carbohydrate metabolism and hormone-mediated plant immune responses. We believe that a general “citrus stress status” can be identified, similar to the “inflammatory response” in animals and consisting of pathways commonly regulated in host responses to HLB, CBCD [24], or CTV [25]. Heat shock proteins and dehydrin preserve protein structural integrity, stabilizing proteins and membranes through chaperone activity [26]. Results presented here confirm that altered expression of these highly interactive proteins could be a key aspect of general stress status.

Secondary metabolism was severely affected, illustrated by the upregulation of genes for biosynthesis of phenylpropanoids, which have potent acidity and peroxynitrite scavenging capacity [27]. However, we focused on two key metabolic pathways: primary metabolism, particularly sucrose and starch metabolism, and hormone biosynthesis, signaling, response, and crosstalk.

### HLB alters source-sink relationships

HLB causes starch accumulation in leaves [1], an observation supported by transcriptomic studies [6], [12]. Our data from young leaves confirm these findings: several starch-related biosynthetic genes such as APL3 and starch branching enzyme were upregulated. This suggests that starch accumulation may start early after CaLas infection and young leaves are the typical CaLas infection sites. Starch metabolism genes (α-glucan phosphorylase 2, α-amylase) were also upregulated in immature leaves as expected. We observed many HLB-downregulated genes involved in light reactions in young leaves, such as genes encoding PSI and PSII subunits of light harvesting complexes I and II. Although mature leaves showed typical HLB symptoms, they remain partially green and actively photosynthesizing. Indeed, infected mature leaves did not drastically decrease light reaction-related transcripts. In fruits, light reaction genes were upregulated by HLB while starch biosynthesis was downregulated. Of particular interest, different isoforms of AGPase had opposite expression patterns (APL1, upregulated; APL3, downregulated), suggesting that different isoforms play tissue-specific roles. Sucrose metabolism was another key pathway affected by the disease (Table 2). Sucrose is the major end product of photosynthetic carbon metabolism and is the predominant carbohydrate transported in phloem sieve tubes from mature leaves to sink organs. Its use relies on invertase activity for hydrolysis into glucose and fructose. Genes for several vacuolar invertases were strongly induced in infected mature fruits. These genes could drastically affect cell osmotic potential and sucrose concentrations in sink tissues, in agreement with the lower concentration of sucrose observed in mature, HLB-infected fruits [28], [29].

**Figure 5:**
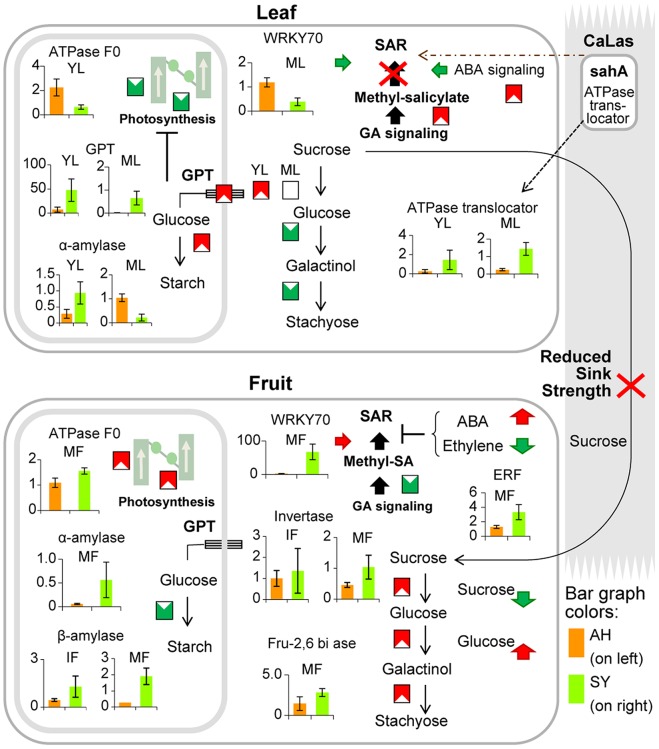
Transcriptional regulation of fruit and leaf metabolism and sugar transport during HLB disease. qRT-PCR data compared AH and SY tissues. White triangles within filled squares indicate up- and down-regulated genes. Arrows pointing up and down likewise indicate increased and decreased metabolite concentration. In each bar graph, AH appears on the right, and SY on the left, where vertical axis units are relative ratios of the test gene and housekeeping gene abundance.

Perhaps related to these changes in invertases, several raffinose metabolism genes were upregulated in fruit, including galactinol synthase, DIN10 (raffinose synthase 6), and stachyose synthase (Figure 3). In leaves with symptoms, sucrose remained more abundant than in healthy leaves, consistent with the unchanged expression of invertase in mature leaves and with our hypothesis that HLB diminishes sucrose flow to sink tissues (Figure 5). Phloem blockage caused by callose is typically detected as symptoms appear and after starch accumulates in leaves [5]. These findings, together with the transcriptomic data presented here, support the hypothesis that the imbalance in sugar partitioning may result not only by a physical phloem dysfunction, but also from transcriptional regulation mechanisms. Sugars such as sucrose and glucose are not only metabolic resources and structural constituents of cells, but they also act as signaling compounds that alter gene expression during plant growth and development [30], [31]. The downregulation of genes related to light reactions observed in young HLB-infected leaves corroborates this hypothesis.

GSEA based on sparse PCA (Figure 1; Dataset S2) showed that HLB affects long distance signaling and transport between sink and source tissues. Functional analysis suggests that concentrations of several ions such as sulfate, selenate, and copper are altered in fruits. In leaves, CaLas induced the expression of genes related to oligopeptide, zinc, and nitrate transport. It is possible that the altered pattern of expression of some of these transport-related genes was partially caused by vascular blockage due to callose accumulation induced by the pathogen [5].

GPT, a gene involved in glucose intracellular transport, was strongly upregulated in infected leaf tissues (Figure 5). This gene is a key regulator of starch accumulation in chloroplasts [32].

GPT plays a key role in providing energy-rich metabolites to pathways supplying plastids with metabolic energy, such as lipid respiration and the energy-rich, glycolytic-intermediate metabolite phosphoenolpyruvate (PEP). We hypothesize that strong GPT upregulation in young leaves represses photosynthesis while inducing starch metabolism pathways in chloroplasts. The abundance of GPT2 mRNA may alter sugar-sensing pathways. Indeed, GPT upregulation may suggest a reduced sucrose flow from leaf to fruit that disrupts the source-sink relationships. Conversely, light reaction transcripts were abundant in ripe infected fruits, a metabolism more characteristic of a source than a sink tissue. Activation of genes involved in sucrose degradation might consequently disrupt the photosynthate gradient between leaves (source) and fruit (sink) (Figure 5). Sucrose loading to the phloem from source leaves involves an apoplastic step. We observed HLB-upregulation of invertase and sucrose synthase genes in young leaves, although further studies are needed to clarify in which cellular compartment. Interestingly, several genes involved in glycolysis and the TCA cycle were upregulated in mature infected leaves. The eventual increase in high-energy compounds induced by HLB supports the hypothesis that CaLas is an energy parasite [11]. However, this must be confirmed by measuring enzyme activities.

### HLB alteration of the hormone-mediated immune response

Current knowledge regarding hormone crosstalk in the plant's immune system has been reviewed by Pieterse et al. [33]. The backbone of the plant immune response consists of salicylic acid (SA), jasmonic acid (JA), and ethylene (ET), assisted by other hormones including ABA, auxins, cytokinins, and brassinosteroids. Recently, the roles of key regulatory proteins in SA-JA crosstalk have been identified. These include MPK4, EDS1, and PAD4 [33]. Interestingly, salicylic acid methyl transferase was induced in young citrus leaves in response to CaLas, although SA signaling and SA-mediated defense responses were scarcely activated (Figure 3). Although SA-mediated defense response genes PIR5 and DIR1 were slightly induced by CaLas in young leaves, transcription of other PR-related proteins was unchanged.

The WRKY family of transcription factors and their responses to biotic and abiotic stress are well known [34]. WRKY70 is a key point of convergence between SA- and JA-dependent defense pathways and is also required for R gene-mediated resistance. WRKY70 and other members were more abundant in mature fruits (Figure 3). It is possible that WRKY upregulation in fruits is due to induction of long distance signaling molecules such as methysalicylate in young leaves. NPR1 plays a central role in SA signal transduction. It acts downstream of EDS1 and regulates SA-mediated expression of GRX480 and WRKY70, proteins that suppress JA-dependent gene expression [35]. A differential role of cytosolic and nuclear NPR1 in regulating JA/ET- and SA-dependent signaling has been demonstrated [36]. In our samples, NPR1 was not induced by HLB in either leaf or fruit tissues. This reinforces the hypothesis of a feeble and insufficient SAR response at the infection site. Interestingly, a homolog of salicylate hydroxylase (sahA) was found in the genome of CaLas [8]. SA breakdown mediated by sahA might help suppress host defenses against pathogen infection (Figure 5).

Increased ethylene bypasses the need for NPR1 in SA-JA crosstalk, resulting in potentiated expression of the SA-responsive marker gene PR-1, which is EIN2-dependent [37]. However, our transcriptomic data showed that both EIN2 and PR-1 expression were unchanged in response to HLB. SY fruits produced less ethylene than AS or healthy ones, independent of maturity [28]. Decreased ethylene in SY fruits could assist retention of green color and fruit aroma. Jasmonic acid is responsible for induced systemic resistance, typically activated in response to necrotroph attacks. Jasmonic acid methyltransferase was induced in young leaves, probably as a response to psyllid attacks. The roles of SCF-COI, JAZ, JA, MYC2, ERF1, and ORA59 have been described by Pieterse et al. [33]. We observed no differences in transcript abundance of these genes, although several jasmonic acid defense response genes were HLB-regulated in all analyzed tissues, especially ripe fruits (Figure 3). Though the SA, JA, and ET response pathways form the backbone of the induced defense signaling network, other hormones clearly modulate crosstalk among them, as demonstrated in *Arabidopsis* [30]. ABA is connected to the SA-JA-ET network, affecting JA biosynthesis, resistance against JA-inducing necrotroph pathogens, and antagonizing the onset of SA-dependent defenses [38]. Interestingly, ABA in SY fruits was higher than in healthy or AS fruits [28]. This agrees with the upregulation of several ABA-responsive genes (GRAM-domain containing protein and CED3; Figure 3). Auxins play a key role in every stage of plant development and the auxin response pathway is connected to the SA-JA-ET signaling network in various ways. The antagonistic effect of SA on auxin signaling is an intrinsic part of SA-dependent resistance against (hemi) biotrophs. Interestingly, auxin-responsive genes were downregulated in young leaves but in immature fruits, GH3.1, GH3.17 and other auxin-responsive proteins were induced, agreeing with the previous IAA analysis of HLB-affected fruits. In particular, auxin-related genes were induced earlier than other hormone-related genes in immature fruit, suggesting that they may be induced at an early stage of HLB. High IAA concentration is associated with cell enlargement and fruit expansion. Localized elevated IAA levels have been linked with development of misshapen fruit [28]. The latter finding may be closely related to HLB-modulation of auxin-responsive genes reported in this study. Brassinosteroids also play a key role in cell expansion and division, differentiation, reproductive development, and fruit ripening. GSEA based on sparse PCA highlighted their key role in fruit symptom appearance. When applied exogenously, they induce broad-spectrum disease resistance [39]. BAK1, involved in brassinosteroid signal transduction, also interacts with receptors that recognize PAMPs such as bacterial flagellin, initiating innate immunity [40]. We observed upregulation of BRS1 signaling in fruits and downregulation of ST1 (involved in brassinosteroid metabolism) in leaves. However, their connections to HLB regulation of SA-ET-JA crosstalk remain to be determined.

### Transcriptional changes in amino acid metabolism

Amino acids play important roles in stress response. Arginine and arginine-rich proteins serve as a reservoir for organic nitrogen in many plants [41]. Proline accumulation has been observed during conditions of abiotic and biotic stresses. Proline biosynthesis may occur in either cytosol or plastids, while arginine biosynthesis is constitutively localized in plastids. The two pathways are connected by a complex regulatory network that allows plants to optimize growth and environmental adaptation [42]. Proline accumulated significantly in HLB-infected leaves [43]. This result partially agrees with a slight increase observed in expression of δ1-pyrroline-5-carboxylate synthetase in HLB-infected leaves, but is not consistent with P5CS enzymatic activity. Argininosuccinate lyase, a key arginine biosynthesis gene, was strongly downregulated in young infected leaves. Early diagnosis of HLB disease is still based on symptom observation followed by PCR-based verification. Novel, in-field detection methods based on simultaneous analysis of induced volatiles, metabolites, and transcripts have been previously proposed [44].

The results presented here can be exploited to develop a test using host genes that change in response to HLB infection which could complement PCR detection of the pathogen. PCR-based detection is problematic due to long incubation times and uneven pathogen distribution in the plant. Rigorous validation will be necessary in controlled environments (infecting the same tissues with different pathogens) to confirm the specificity of potential biomarkers for HLB disease. The discovery of pre-symptomatic biomarkers, along with cost-effective and robust methods, promises not only more effective ways to detect primary sources of infection, but also for validation of the effects of the therapeutic strategy. If a therapeutic strategy is effective, the biomarkers should revert to expression characteristic of healthy tissues. In-field analysis of these transcripts can be enhanced through development of novel devices such as LFM technology to enable rapid, hybridization-based nucleic acid detection using an easily visualized colorimetric signal.

Based on these findings, we propose several possible short-term therapeutic approaches for already infected trees. The first approach aims to restore a normal source-sink relationship by modulating the expression of key genes in young leaves such as GPT and invertase. The application of compounds having a negative effect such as potassium nitrate (KNO_3_), GA_4_, 6-benzyladenine [43] might confer a beneficial effect countering starch accumulation in plastids and the decrease in photosynthesis (Figure S9). Data from the Genevestigator database suggest that these compounds downregulate the expression of GPT in Arabidopsis leaves [45].

The second approach could focus on boosting the plant immune response using arginine and enhancing the expression of PR proteins. L-Arginine is the precursor of nitric oxide (NO). Endogenous NO concentrations were positively correlated with PAL, PPO, CHI, and GLU activities in response to *Botrytis cinerea* in tomato fruits [46]. Since auxin-responsive genes are the earliest induced hormone-related genes when fruits are still immature, a worthwhile experiment would be a test of auxin inhibitor compounds targeted to the small fruits of infected trees. A forth possible strategy might use sugar sensing by applying sucrose. This would involve Zinc Finger, AP2-EREBP and WRKY70 to upregulate detoxifying genes such as GSTF8 and FSD8 known to play roles in xenobiotic and oxidative stress [47]. The combination of sucrose with atrazine might be investigated since previous data have suggested important synergistic interactions between the two compounds for xenobiotic resistance through ROS signaling induction [48].

### Conclusions

We have presented a broad picture of the metabolic changes in mature and immature leaves and fruits (both sink and source tissues). In addition, we have identified early and late responses to HLB infection by comparing apparently healthy, infected but asymptomatic, and symptomatic trees. The timing of changes suggested which HLB-regulated pathways could be involved in causes or effects of the disease. The field analysis of gene expression was critical to distinguish the complexity of signalling networks involved in plant-microbe interactions against the background noise of other environmental and agronomic factors. This level of analysis is essential to complement controlled studies limited to only a few factors. Our results highlight how the pathogen differentially affects sugar and starch metabolism in young and mature leaves and fruits. The upregulation of GPT in young leaves was key to inducing starch accumulation, with a consequent decrease in photosynthesis. The upregulation of sucrose metabolism added to the source-sink metabolic dysfunction that, in our opinion, is the most probable cause of the disease. The important HLB-induced changes in hormone networks surely also play a pivotal role in the metabolic disorder. Upregulation of some key genes in jasmonic acid synthesis could confound the salicylic acid response, considered the appropriate counterattack to biotrophs. This explains the lack of bacterial clearance leading to a chronic infection. Consistent with this hypothesis, WRKYs (i.e. WRKY70) were unexpectedly expressed at higher levels in fruit, a tissue not believed to be a primary site of infections. These findings may lead to improved detection methods based on host responses, and enable the validation of short-term therapeutic strategies. Using hormones and other small molecules holds promise to reverse the metabolic dysfunction and improve the innate immune response to this devastating disease of *Citrus*.

## Materials and Methods

### Plant material and experimental design

The transcriptome expression analysis compared the expression of each transcript in the symptomatic (SY) category with apparently healthy (AH), asymptomatic, (AS) and healthy control tissues (CO) for each of the four analyzed tissues from ‘Valencia’ sweet orange (*C. sinensis* L. Osb.): immature leaves (YL) in apical shoots (length >3 cm, open but not fully expanded), mature leaves (ML) which were fully expanded, immature fruit peel (IF) which were green and not fully expanded, and mature fruit peel (MF) which were fully expanded (Table 1). The four HLB categories were defined based on phenotype and presence of the pathogen. SY and AS samples were collected from the same infected trees located at the USHRL-USDA farm in Fort Pierce (St. Lucie County, FL). All trees at this location tested positive for the pathogen by PCR at the time of collection. AS and SY, the first two categories of fruit peel, were collected from trees with typical HLB disease symptoms on leaves (blotchy mottle and chlorosis) and fruit (small, green, and irregular in shape). Trees were tested by PCR assay for the presence of CaLas using petioles from four to six leaves collected from different areas in the canopy. AH trees at the same location were PCR-positive but did not display symptoms at the time of sampling. The fourth category was healthy fruit from “Valencia” PCR-negative trees at a disease-free location, the Citrus Research and Education Center (Lake Alfred, FL). A pool of five to ten different leaves or fruits were collected from each of five different trees per treatment group, representing five biological replicates. Tissue samples were stored at −20°C for PCR detection of CaLas. Fruit peel segments were cut and mixed, immediately frozen in liquid nitrogen, and stored at −80°C. Juice sacs were removed before extraction.

### PCR detection of CaLas

Petioles and peduncles were ground in liquid nitrogen with a mortar and pestle and 100 mg ground tissue was used for DNA extraction. DNA was extracted using the Plant DNeasy® Mini Kit (Qiagen, Valencia, CA) according to manufacturer's instructions, yielding 20 to 30 ng DNA per extraction. Real-time PCR assays were performed using primers HLBas (5′- TCGAGCGCGTATGCAATACG -3′) and HLBr (5′- GCGTTATCCCGTAGAAAAAGGTAG -3′) and probe HLBp (5′- AGACGGGTGAGTAACGCG -3′) [49]. Amplifications were performed using an ABI 7500 real-time PCR system (Applied Biosystems, Foster City, CA) and the QuantiTect Probe PCR Kit (Qiagen) according to manufacturer's instructions. All reactions were carried out in duplicate in a 20-µL reaction volume using 5 uL cDNA reaction. Plants or fruits were considered PCR-positive when Ct (cycle threshold) values were below 32.

### RNA extraction

Total RNA from each biological replicate was isolated using phenol/chloroform/isoamylalcohol (25∶24∶1) extraction followed by two extractions with chloroform/isoamylalcohol and precipitation of RNA with isopropanol at −20°C overnight [12]. RNA was further purified using the RNeasy MinElute Cleanup kit (Qiagen) according to the manufacturer's instructions. RNA concentrations were determined using a NanoDrop ND-1000 spectrophotometer (NanoDrop Technologies, Wilmington, DE). RNA quality and purity were assessed by an Agilent Bionalyzer (Folsom, CA).

### cDNA library construction and high throughput sequencing

RNA from the five biological replicates was equally pooled to 10 µg and then used to construct one cDNA library for each of the four HLB status categories for each tissue. The cDNA libraries were constructed following the Illumina mRNA-sequencing sample preparation protocol (Illumina Inc., San Diego, CA). Final elution was performed with 16 µL RNase-free water. The quality of each library was determined using a BioRad Experion (BioRad, Hercules, CA). Each library was run as an independent lane on a Genome Analyzer II (Illumina, San Diego, CA) to obtain read lengths of up to 85 bp per paired end.

### Sequence data processing and analysis

For the 16 cDNA libraries, a total of 889 million 85 bp paired-end raw reads were obtained with the Illumina Genome Analyzer II. These reads were trimmed to remove low-quality regions using custom scripts. The trimmed paired-end reads from each library were aligned to the *Citrus sinensis* genome scaffolds using Bowtie [50] and TopHat [51]. The *C. sinensis* genome sequence data were produced by the US Department of Energy Joint Genome Institute (http://www.jgi.doe.gov) in collaboration with the user community, as a collaborative effort led by 454 Life Sciences, University of Florida and JGI [22], http://www.phytozome.net/citrus

The Cufflinks software suite was used for reference annotation-based transcript assembly [52] to identify expressed genes and transcript isoforms already annotated on the 12,574 *C. sinensis* genome v.1 assembly scaffolds [23] and to discover previously unannotated genes and splice variants. This generated new transcriptome sequences to which all of the Illumina reads were mapped with the BWA short read aligner [53]. A table of raw counts, generated with a custom script, was then used as input for differential gene expression analysis.

A list of differentially regulated transcripts was obtained for three pairwise comparisons (CO vs. SY, AH vs. SY, AS vs. SY) for each tissue. For each pairwise comparison, the raw count data was normalized to control for different sequencing depths across samples, using the DESeq Bioconductor package [54].

RNA-Seq and differential expression data were deposited in the SRA and GEO databases of NCBI, with accession number SRP022979.

### Sparse principal component analysis

A sparse principal component analysis (sPCA) technique [55] was used to determine which genes contribute the most to the differences across the SY, AS, and AH samples. This PCA-based approach was chosen to avoid the complexity of a multiple pairwise comparison approach, and to determine which genes explain the most variation across the four tissue samples simultaneously.

The sPCA technique was applied across the variance-stabilized, normalized counts of the SY, AS, and AH samples for each tissue (leaf or fruit) and maturity (young/immature or mature). The normalization approach of DESeq was also used to correct for library size differences. A variance-stabilizing transform (VST) from the DESeq Bioconductor package [7] was used to correct for the higher variance of many genes due to the count nature of the data. For each sample, the thresholding parameter was increased until 40% of the variance was explained, resulting in different numbers of genes with non-zero coefficients in each sample given in Table 3.

**Table 3:** Numbers of genes with non-zero coefficients in each tissue type in Sparse Principal Component Analysis.

tissue type	number of genes with non-zero coefficients
IF	10361
MF	8002
YL	3920
ML	10419

Preliminary validation of this novel application of the sPCA technique to unreplicated VST RNA-Seq data was performed initially, as tuning the thresholding parameter was not possible against other criteria.

Sparse PCA was used to identify genes with expression that most strongly distinguishes three of the samples: AH, AS, and SY; CO was excluded. Using the list of genes from sparse PCA, a gene set enrichment analysis was performed, considering only AH vs. SY in the four tissues, restricted to plant-specific GO terms in the file ATH_GO_GOSLIM.txt from www.arabidopsis.org.

Sequencing using the Illumina platform was carried out at the UC Davis Genome Center, DNA Technology Core Facility. Processing and assembly of raw sequence reads and sPCA analyses were carried out at the UC Davis Genome Center, Bioinformatics Core Facility.

### Functional categorization of predicted transcripts

Blast2GO [56] was used to assign annotations and Gene Ontology (GO) terms to the predicted transcripts of *Citrus sinensis*, produced by the Cufflinks suite. *Arabidopsis* orthologs were determined for transcripts by blastx (e-value <10^–4^) to the TAIR database of predicted proteins in *Arabidopsis* (TAIR10_pep_20101028; [57]). Blastx output was processed using custom scripts to calculate the best correspondence between individual citrus assembly sequences and *Arabidopsis* proteins, based on alignments over the entire length of each sequence. Lists of predicted transcripts that were differentially expressed at a significant level (*p*<0.01, absolute value of log fold change >1.5) in the pairwise comparisons. These were used as input for one-tailed Fisher's Exact Test in Blast2GO to identify enriched GO terms. Functions of differentially expressed genes (as *Arabidopsis* orthologs) were visualized using MapMan [58]. Gene set enrichment analysis was also performed using Pathexpress [59], again for *Arabidopsis* orthologs of differentially expressed transcripts (*p*<0.01, absolute value of log fold change >1.5). The PageMan visualization tool [60] was also used for GSEA with Wilcoxon test, no correction and 1.0 as ORA cutoff.

### Protein-protein interaction (PPI) network

A predicted protein interactome was constructed for *Citrus* based on PPIs in *Arabidopsis* [61] for each of the four tissues studied. Networks were identified and visualized using Cytoscape software [62]. Nodes of the network represented proteins encoded by HLB-regulated genes (fold ratio >1.0 and <−1.0; comparison between SY and AH) and their functional partners in the predicted pairwise interaction network.

### Real time TaqMan® PCR system

Real time TaqMan® PCR analysis was conducted to validate the RNA-Seq data. Three biological replicates of five to ten fruits or leaves for each tissue type from trees of each HLB status (AH, AS, SY, CO) were pooled. For each target gene, PCR primers and a TaqMan® probe were purchased as an assay mix from Applied Biosystems (Foster City, CA). DNase treatment and cDNA synthesis were performed in a combined protocol following the Quantitect Reverse Transcription Kit (Qiagen) instructions. A standard curve to determine the linearity of amplicon quantity vs. initial cDNA quantity was generated for each gene. Amplifications used 25 ng cDNA in a 20 µL final volume with TaqMan Universal PCR Master Mix and Taqman Assay ABI mixes (Applied Biosystems). Amplications were performed on a StepOne Real Time PCR system (Applied Biosystems) using standard amplification conditions: 1 cycle of 2 min at 50°C, 10 min at 95°C; 40 cycles of 15 s at 95°C; and 60 s at 60°C. All PCR reactions were performed in duplicate. Fluorescent signals were collected during the annealing temperature and C_T_ values extracted with a threshold of 0.04 and baseline values of 3 to 10. *Citrus sinensis* elongation factor 1 alpha (EF-1α, accession AY498567) was used as an endogenous reference and ΔΔ*C*_T_ was calculated by subtracting the average EF-1α *C*_T_ from the average *C*_T_ of the gene of interest. Real time TaqMan® PCR analysis was conducted to assess CTV presence in fruit and leaf samples. Primers designed on CTV CP reference sequence T36 (M76485) were used with the same protocol used for the analysis of citrus genes.

## Supporting Information

Figure S1 **Transcriptome principal component analysis of 16 different citrus samples.**
(PDF)

Figure S2 **PageMan gene set enrichment analysis.**
(PDF)

Figure S3 **Gene expression changes caused by HLB in four tissues seen in MapMan metabolism overview.**
(PDF)

Figure S4 **Expression changes caused by HLB in transcripts encoding starch and sucrose metabolism.**
(PDF)

Figure S5 **Visualizations of gene expression changes caused by HLB in small carbohydrate metabolism and hormonal signaling.**
(PDF)

Figure S6 **HLB-modulation of arginine and proline pathways.**
(PDF)

Figure S7 **Predicted interaction networks between proteins encoded by HLB-regulated genes.**
(PDF)

Figure S8 **Overview of principal transcriptional changes induced by HLB.**
(PDF)

Figure S9 **Proposed short-term therapeutic strategy to mitigate the source-sink metabolic dysfunction.**
(PDF)

Dataset S1 **Predicted transcripts and differential expression.** (A) *Arabidopsis* orthologs corresponding to citrus HLB-regulated transcripts in three pairwise comparisons for each tissue. (B) HLB-differentially regulated genes grouped by pathways for MapMan analysis. Fold ratio for three pairwise comparisons (CO_vs_SY, AH_vs_SY, AS_vs_SY) for each of the four tissues are indicated.
(XLSX)

Dataset S2 **GO categories of transcripts significantly affected by HLB, based on sparse principal component analysis.**
(XLSX)

Dataset S3 **qRT-PCR data for 109 transcripts in the four HLB categories for each tissue.**
(XLSX)

Dataset S4 **HLB-regulated genes in citrus encoding interactive proteins.**
(XLSX)

Table S1 Pathway enrichment analysis of mature leaf responses to HLB, CTV, and CBCD using Pathexpress.
(DOC)
