# Supplementary material for: Gene Regulatory Networks Elucidating Huanglongbing Disease Mechanisms
Source: PLoS One. 2013 Sep 25;8(9):e74256. doi: 10.1371/journal.pone.0074256 (PMC3783430; doi:10.1371/journal.pone.0074256)
Supplement: Figure S1 — Transcriptome principal component analysis of 16 different citrus samples. (PDF) [file pone.0074256.s001.pdf]

A

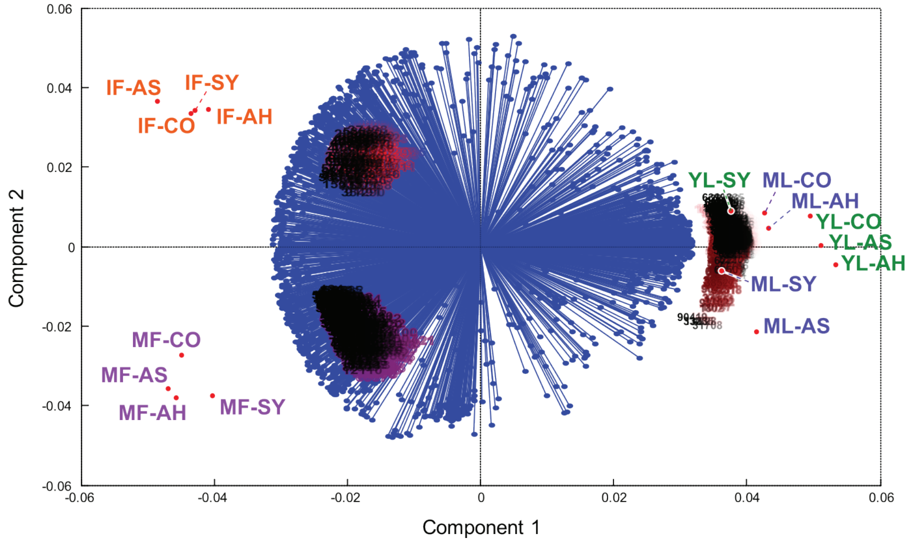


B

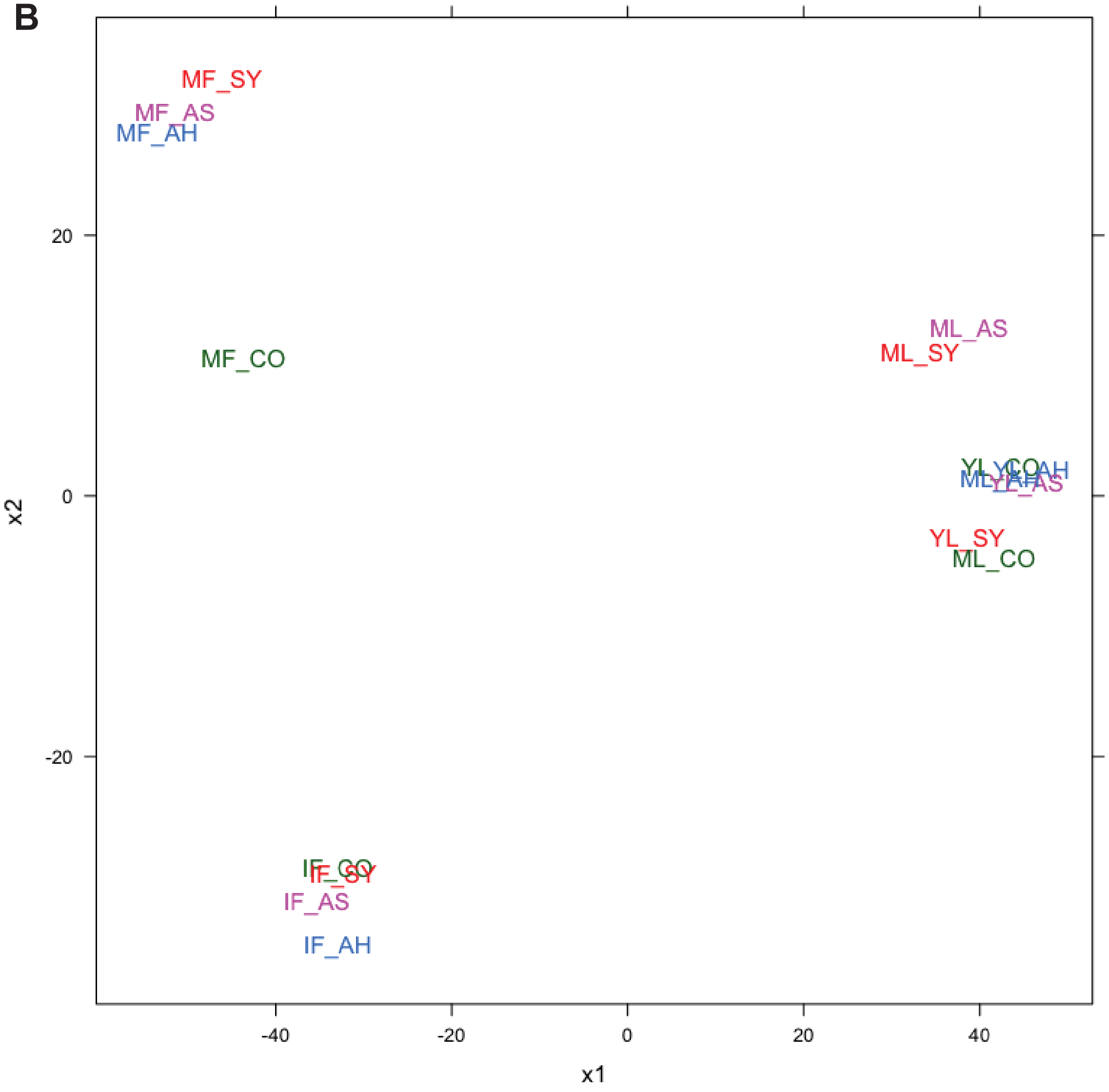


**Figure S1.** Transcriptome principal component analysis of 16 different citrus samples: control healthy, CO; apparently healthy, AH; asymptomatic, AS; symptomatic, SY; immature fruit, IF; mature fruit, MF; young leaves, YL; mature leaves, ML. Panel (A) was generated by Principal component analysis (following a method different from sparse PCA described in Materials and Methods). Panel (B) was generated based on pairwise comparisons using the DEseq package of R. To generate the graph in (A), within-sample normalization process was applied to each sample to calculate the ratio of each predicted transcript. A principal component analysis of all 16 citrus categories was performed. To examine which transcripts contribute most to each class, two criteria were employed. First, the mean values of the loading vector lengths (strengths) of all transcripts were calculated and 80% of the mean value was used as the threshold for strength screening. Secondly, the directional similarity between each transcript and each class was determined and 0.98 (i.e, the cosine value of 10°) was set as the threshold value for similarity screening. Thus, the sum of the ratios of all transcripts was 1 and each transcript had its own count ratio between 0 and 1. For each sample, the transcripts were first sorted from high to low. Then the highest-ranking transcripts with a cumulative ratio of 25% of the total were retained for further analysis. This narrowed the analysis to a small number of transcripts. By integrating all of these for the four classes, a list of transcripts of interest was generated. In this list, some were shared by multiple classes while others were only observed in one class. Principal component analysis was then applied to the ratio matrix of this list to examine the contribution of each transcript to the separation of the classes.
