# Supplementary figures and images for: Gene Regulatory Networks Elucidating Huanglongbing Disease Mechanisms
Source: PLoS One. 2013 Sep 25;8(9):e74256. doi: 10.1371/journal.pone.0074256 (PMC3783430; doi:10.1371/journal.pone.0074256)

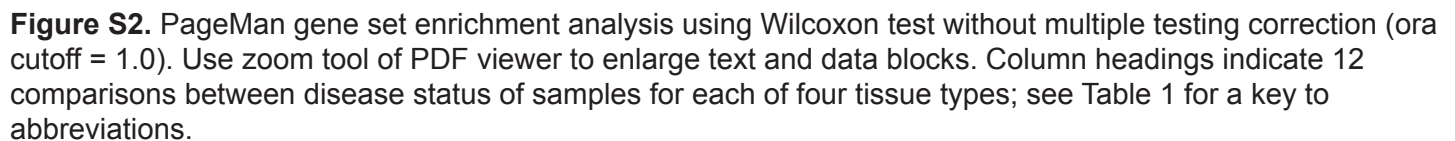

Supplement: Figure S2 — PageMan gene set enrichment analysis. (PDF) [file pone.0074256.s002.pdf]
