# Supplementary material for: Gene Regulatory Networks Elucidating Huanglongbing Disease Mechanisms
Source: PLoS One. 2013 Sep 25;8(9):e74256. doi: 10.1371/journal.pone.0074256 (PMC3783430; doi:10.1371/journal.pone.0074256)
Supplement: Figure S3 — Gene expression changes caused by HLB in four tissues seen in MapMan metabolism overview. (PDF) [file pone.0074256.s003.pdf]

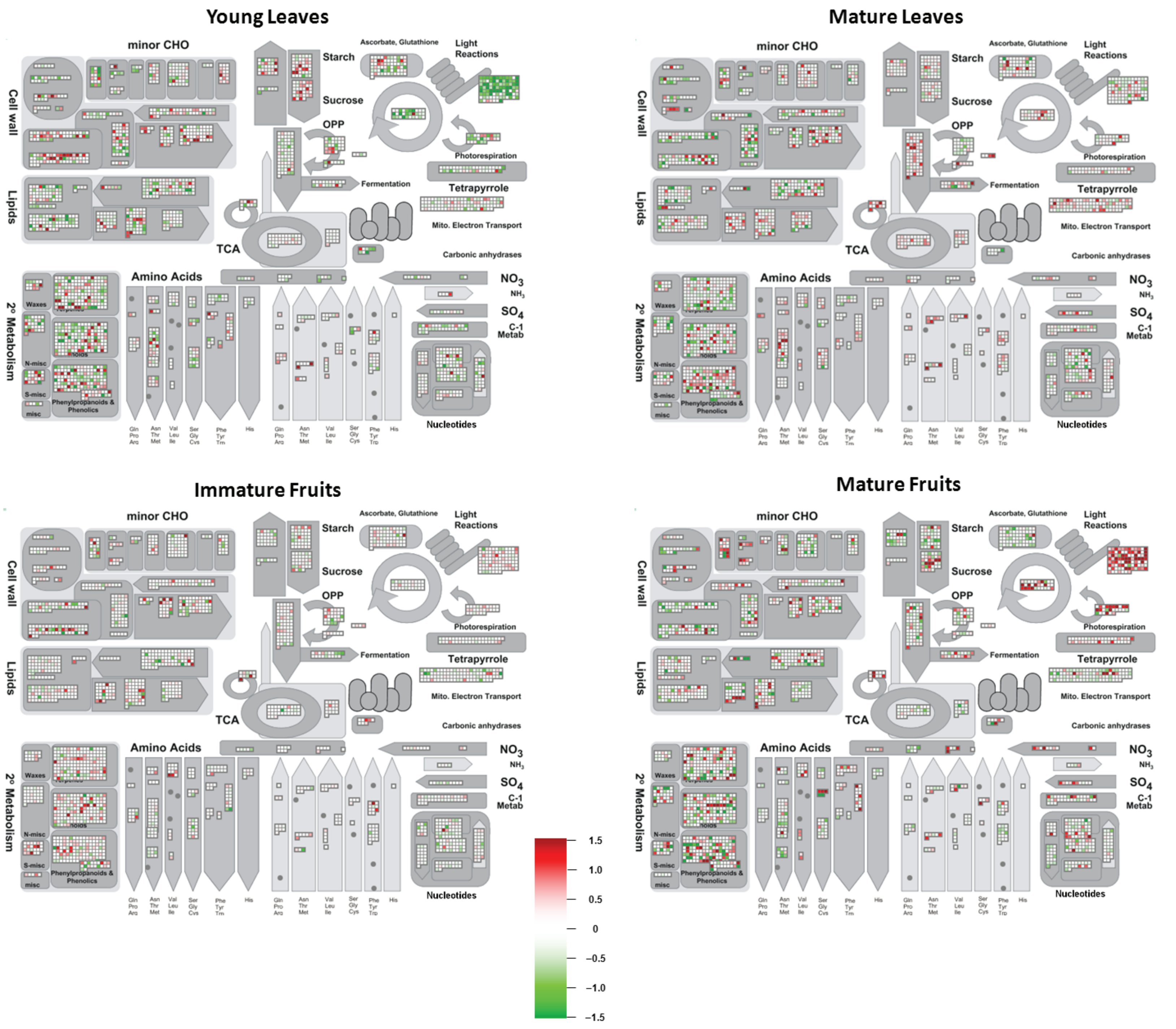

Figure S3. Gene expression changes caused by HLB in four tissues seen in the metabolism overview of MapMan.
