# Supplementary material for: Gene Regulatory Networks Elucidating Huanglongbing Disease Mechanisms
Source: PLoS One. 2013 Sep 25;8(9):e74256. doi: 10.1371/journal.pone.0074256 (PMC3783430; doi:10.1371/journal.pone.0074256)
Supplement: Figure S4 — Expression changes caused by HLB in transcripts encoding starch and sucrose metabolism. (PDF) [file pone.0074256.s004.pdf]

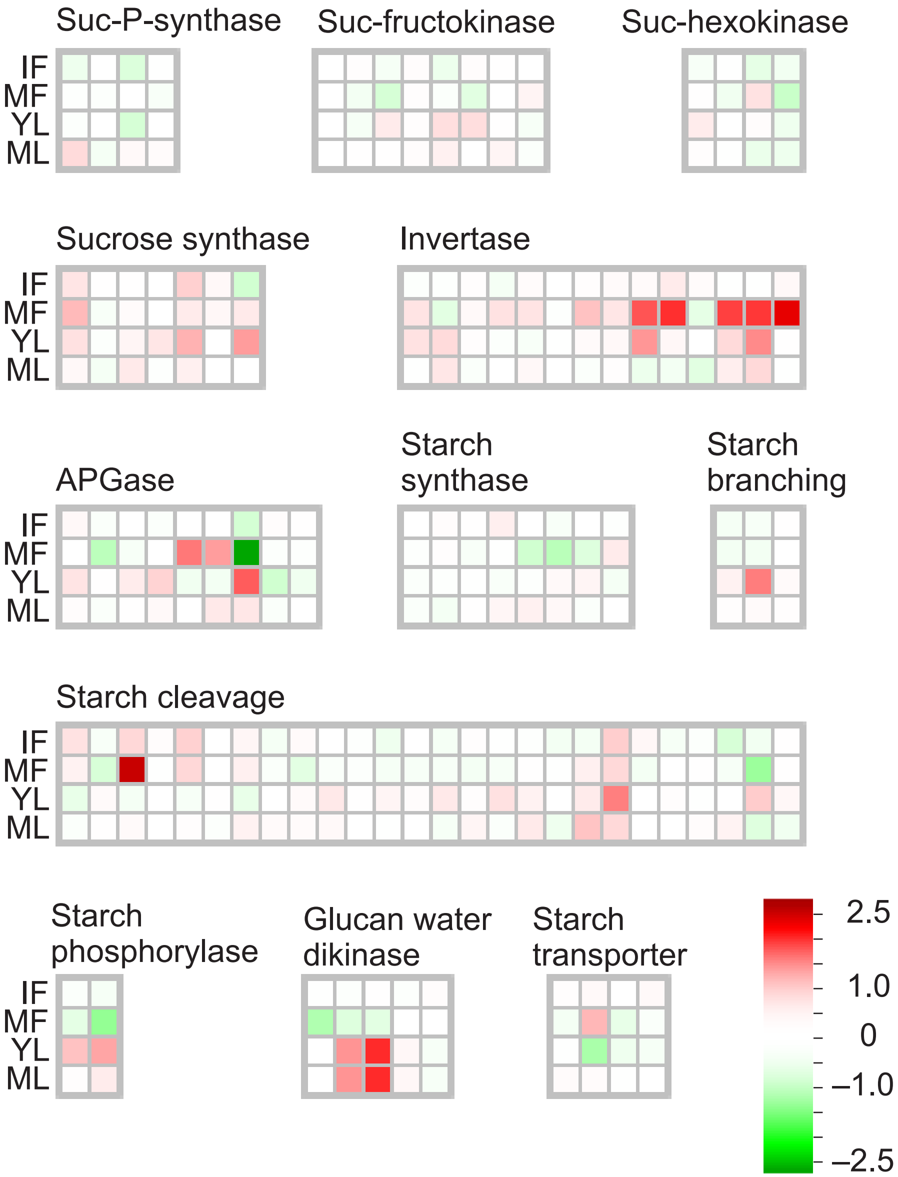

Figure S4. Expression changes caused by HLB in transcripts encoding starch and sucrose metabolism, comparing symtomatic to apparently healthy samples. Tissue types are indicated on the left margin of each block of data.
