# Supplementary material for: Gene Regulatory Networks Elucidating Huanglongbing Disease Mechanisms
Source: PLoS One. 2013 Sep 25;8(9):e74256. doi: 10.1371/journal.pone.0074256 (PMC3783430; doi:10.1371/journal.pone.0074256)
Supplement: Figure S5 — Visualizations of gene expression changes caused by HLB in small carbohydrate metabolism and hormonal signaling. (PDF) [file pone.0074256.s005.pdf]

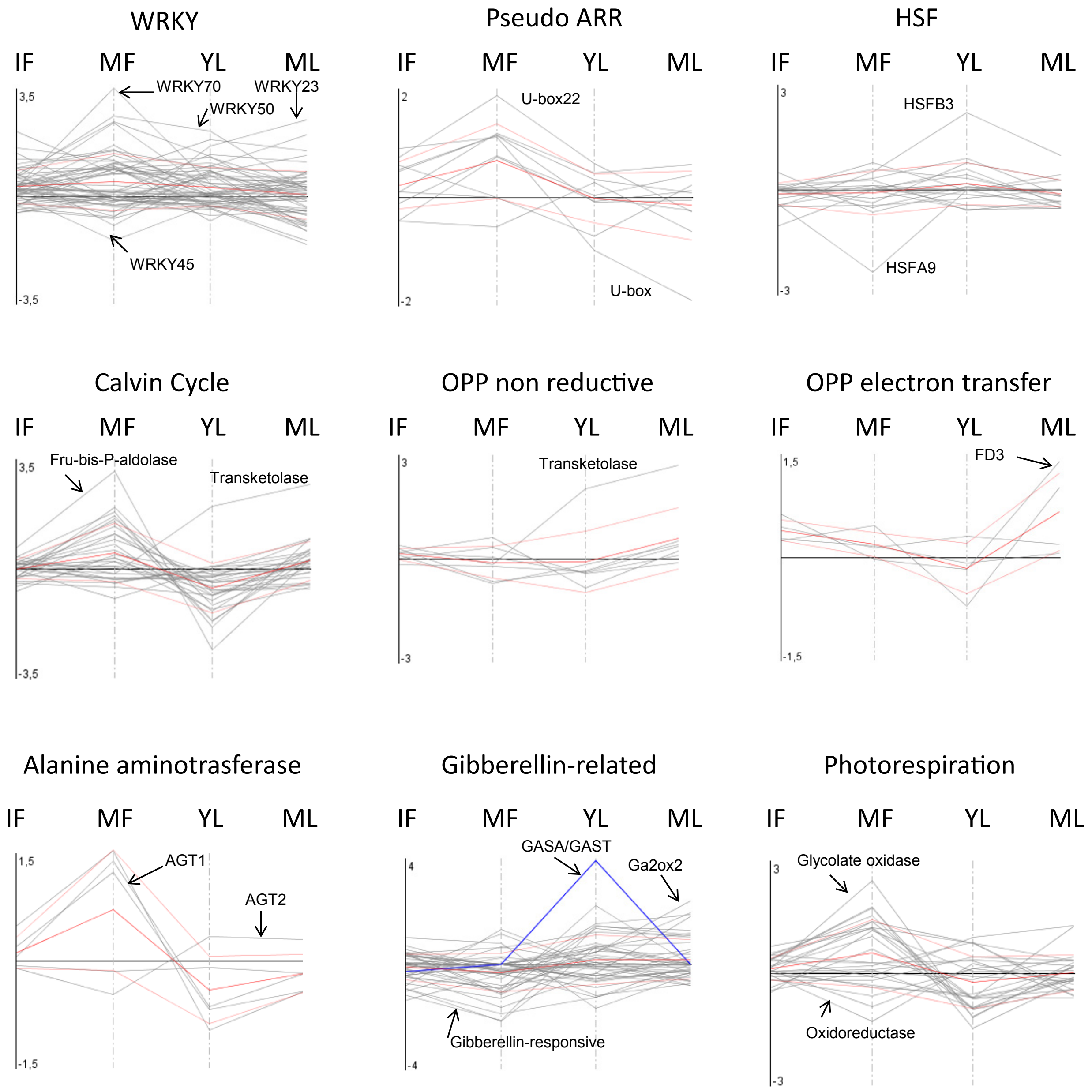

Figure S5. Expression changes caused by HLB in a subset of transcripts with opposite expression patterns in the four tissues (see Table 1 for a key to abbreviations). Tissue types are indicated as column headings above each graph.
