# Supplementary material for: Gene Regulatory Networks Elucidating Huanglongbing Disease Mechanisms
Source: PLoS One. 2013 Sep 25;8(9):e74256. doi: 10.1371/journal.pone.0074256 (PMC3783430; doi:10.1371/journal.pone.0074256)
Supplement: Figure S6 — HLB-modulation of arginine and proline pathways. (PDF) [file pone.0074256.s006.pdf]

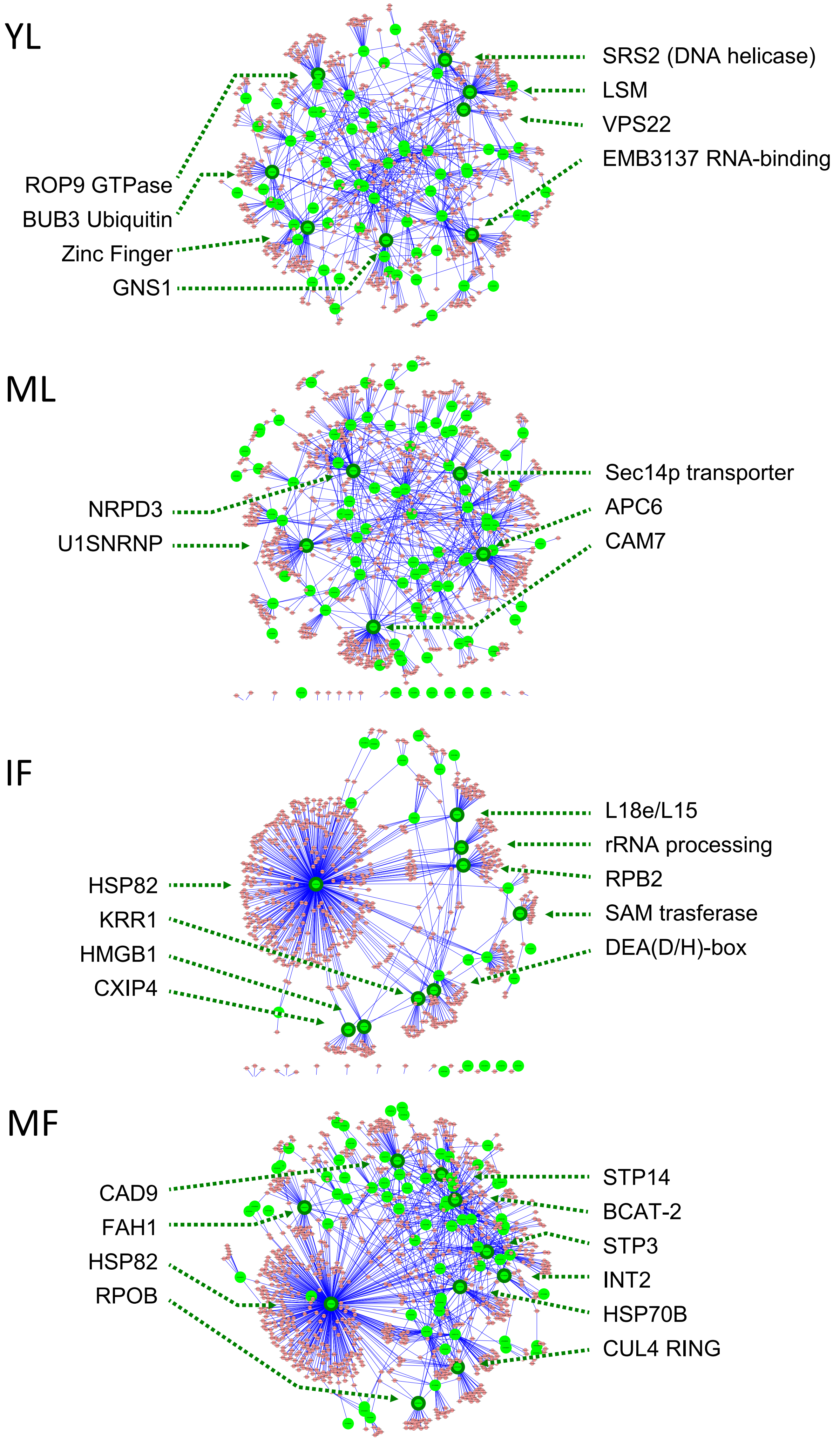


**Figure S6.** Predicted interaction networks between proteins encoded by HLB-regulated genes based on the *Arabidopsis* knowledgebase (Geisler-Lee *et al.*, 2007). Identifiers of orthologs in *Arabidopsis* representing individual proteins can be viewed by using the zoom tool in PDF viewer.; see Table 1 for a key to abbreviations used in left-hand column.
