# Supplementary material for: Gene Regulatory Networks Elucidating Huanglongbing Disease Mechanisms
Source: PLoS One. 2013 Sep 25;8(9):e74256. doi: 10.1371/journal.pone.0074256 (PMC3783430; doi:10.1371/journal.pone.0074256)
Supplement: Figure S7 — Predicted interaction networks between proteins encoded by HLB-regulated genes. (PDF) [file pone.0074256.s007.pdf]

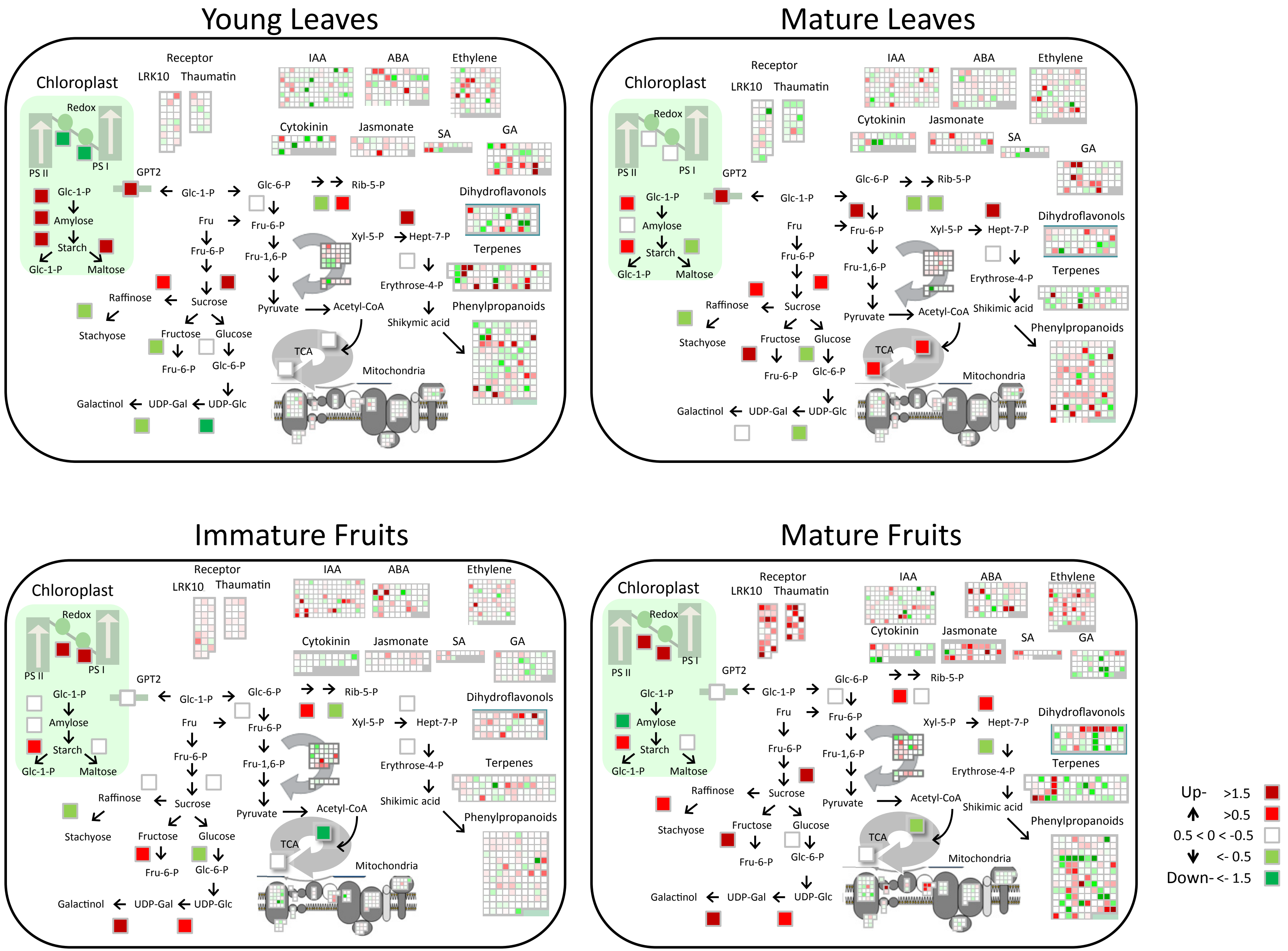

Figure S7. Overview of principal transcriptional changes induced by HLB (those in Fig. 2 along with those associated with hormone and secondary metabolism) induced by HLB in four tissues.
