# Supplementary material for: Gene Regulatory Networks Elucidating Huanglongbing Disease Mechanisms
Source: PLoS One. 2013 Sep 25;8(9):e74256. doi: 10.1371/journal.pone.0074256 (PMC3783430; doi:10.1371/journal.pone.0074256)
Supplement: Figure S8 — Overview of principal transcriptional changes induced by HLB. (PDF) [file pone.0074256.s008.pdf]

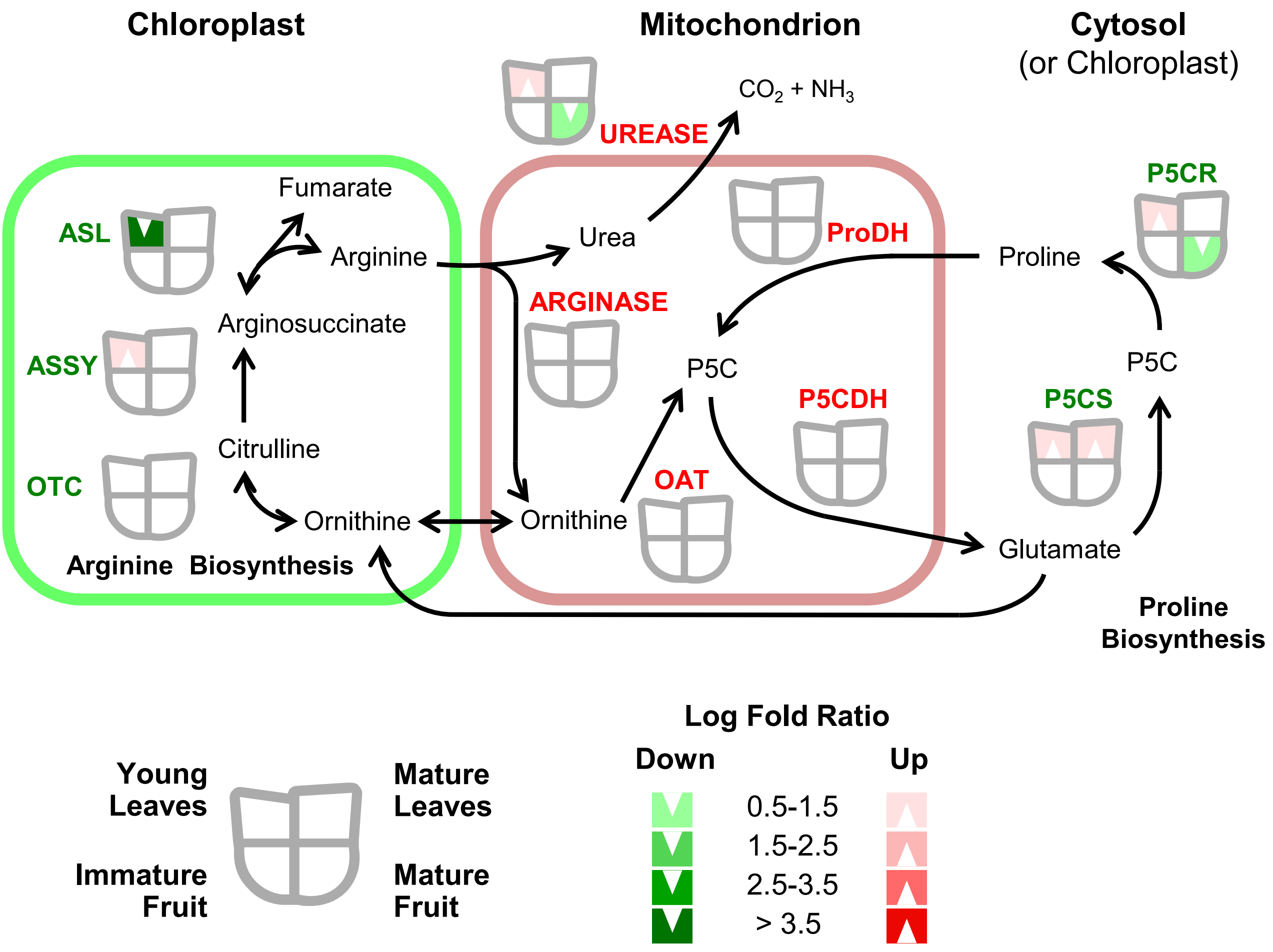

Figure S8. HLB-modulation of arginine and proline pathways. Color values indicate transcript abundance, in symptomatic compared to apparently healthy samples, are indicated by the same convention as in Fig. 2.
