# Supplementary material for: Gene Regulatory Networks Elucidating Huanglongbing Disease Mechanisms
Source: PLoS One. 2013 Sep 25;8(9):e74256. doi: 10.1371/journal.pone.0074256 (PMC3783430; doi:10.1371/journal.pone.0074256)
Supplement: Figure S9 — Proposed short-term therapeutic strategy to mitigate the source-sink metabolic dysfunction. (PDF) [file pone.0074256.s009.pdf]

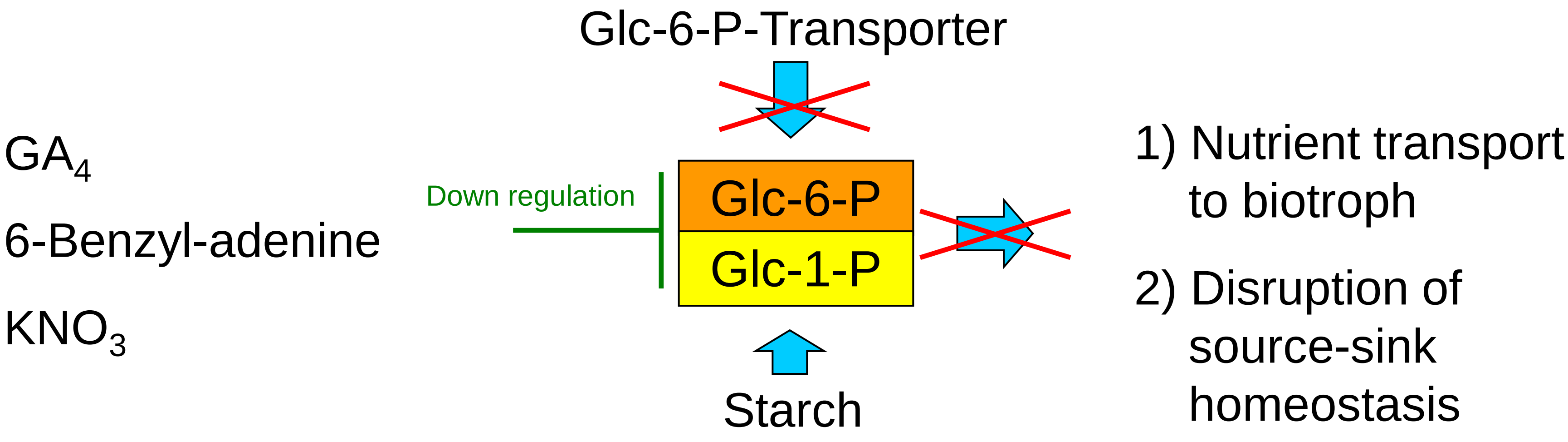

Data from Genevestigator database (https://www.genevestigator.com/gv/):

KNO_3 — KAT-00417 / ArrayExpress-annotation

GA_4 — AT-00409 / GEO-annotation

6-Benzyl-adenine — AT-00156 / GEO-annotation

Figure S9. Proposed short-term therapeutic strategy to reverse source-sink dysfunction using regulators of glucose transporter
