# Supplementary material for: Gene Regulatory Networks Elucidating Huanglongbing Disease Mechanisms
Source: PLoS One. 2013 Sep 25;8(9):e74256. doi: 10.1371/journal.pone.0074256 (PMC3783430; doi:10.1371/journal.pone.0074256)
Supplement: Table S1 — Pathway enrichment analysis of mature leaf responses to HLB, CTV, and CBCD using Pathexpress. (DOC) [file pone.0074256.s014.doc]

**Table S1.** Differentially regulated* pathways in mature leaf tissues in response to HLB, CTV, and Citrus Canker.

| Pathway | HLB | CTV | Citrus Canker |
| --- | --- | --- | --- |
| Starch and sucrose metabolism | 2*10^-4^ | n.s. | 0.02 |
| Phenylpropanoid biosynthesis | 0.01 | n.s. | 0.05 |
| Indole and ipecacalkaloid biosynthesis | 0.02 | n.s. | n.s. |
| Glycerolipid metabolism | 0.04 | n.s. | n.s. |
| Glycolysis / Gluconeogenesis | 0.05 | 0.02 | n.s. |
| Carbon fixation | 0.09 | n.s. | n.s. |
| Pentose phosphate pathway | n.s. | 0.02 | n.s. |
| Glutathione metabolism | n.s. | 0.04 | n.s. |
| Ascorbate and Aldarate Metabolism | n.s. | 0.04 | n.s. |
| Linoleic Acid Metabolism | n.s. | 0.05 | n.s. |
| Alkaloid biosynthesis I | n.s. | n.s. | 2*10^-3^ |
| Pentose and glucuronate | n.s. | n.s. | 4.7*10^-3^ |
| Nucleotide sugar metabolism | n.s. | n.s. | 7.8*10^-3^ |

* Enrichment based on genes with log fold ratio < -1.5 and > 1.5; p-values are shown for CTV, CBCD, found using Pathexpress web tool: p< 0.1 considered to be significantly regulated; "n.s." = "not significant."
